# Nocturnal “humming” vocalizations: adding a piece to the puzzle of giraffe vocal communication

**DOI:** 10.1186/s13104-015-1394-3

**Published:** 2015-09-09

**Authors:** Anton Baotic, Florian Sicks, Angela S. Stoeger

**Affiliations:** Department of Cognitive Biology, University of Vienna, Althanstr. 14, 1090 Vienna, Austria; Berlin Tierpark, Am Tierpark 125, 10319 Berlin, Germany

**Keywords:** *Giraffa camelopardalis*, Giraffe, Hum, Vocalization, Call, Acoustic, Communication

## Abstract

**Background:**

Recent research reveals that giraffes (*Giraffa camelopardalis* sp.) exhibit a socially structured, fission–fusion system. In other species possessing this kind of society, information exchange is important and vocal communication is usually well developed. But is this true for giraffes? Giraffes are known to produce sounds, but there is no evidence that they use vocalizations for communication. Reports on giraffe vocalizations are mainly anecdotal and the missing acoustic descriptions make it difficult to establish a call nomenclature. Despite inconclusive evidence to date, it is widely assumed that giraffes produce infrasonic vocalizations similar to elephants. In order to initiate a more detailed investigation of the vocal communication in giraffes, we collected data of captive individuals during day and night. We particularly focussed on detecting tonal, infrasonic or sustained vocalizations.

**Findings:**

We collected over 947 h of audio material in three European zoos and quantified the spectral and temporal components of acoustic signals to obtain an accurate set of acoustic parameters. Besides the known burst, snorts and grunts, we detected harmonic, sustained and frequency-modulated “humming” vocalizations during night recordings. None of the recorded vocalizations were within the infrasonic range.

**Conclusions:**

These results show that giraffes do produce vocalizations, which, based on their acoustic structure, might have the potential to function as communicative signals to convey information about the physical and motivational attributes of the caller. The data further reveal that the assumption of infrasonic communication in giraffes needs to be considered with caution and requires further investigations in future studies.

**Electronic supplementary material:**

The online version of this article (doi:10.1186/s13104-015-1394-3) contains supplementary material, which is available to authorized users.

## Findings

### Background

A lion “roars”, a dog “barks”, an elephant “trumpets”, but what does a giraffe sound like? Indeed, to date, our knowledge about giraffe (*Giraffa camelopardalis* sp.) vocal communication is limited due to a lack of systematic scientific investigations, while other aspects of giraffe behaviour have now received more research attention [1–4].

In contrast to the prevalent opinion that giraffe herds are loose amalgamations of non-bonded individuals, recent behavioural research on long-term data indicates that they possess a structured, fission–fusion social system in which herd composition is apparently based upon social associations that often reflect kinship [5–7]. Social affiliation and attachment among individuals have also been observed in captive giraffes [8–10].

Species with a fission–fusion society (such as the African elephant *Loxodonta africana*, African buffalo *Syncerus caffer*, spotted hyaena *Crocuta crocuta* and the chimpanzee *Pan troglodytes*) often exhibit a sophisticated vocal communication system to facilitate social dynamics [11–16]. Important vocalization types include long-distance contact calls that convey individual identity [11, 17–19] as well as vocalizations to confirm and strengthen social bonding when reunited after temporary separation.

Although giraffes do have a well-developed larynx and laryngeal nerves [20–22], it was long suggested that due to the long neck, giraffes might have problems to produce an air-flow of sufficient velocity to induce self-sustained vocal fold vibrations [20]. Notwithstanding, giraffes are, in principle, capable of producing sounds [23]. On YouTube there is a video of a newborn calf at a zoo emitting loud bellows while being restrained by keepers to examine its health state [24]. Giraffes do not seem to use vocalizations regularly, but they have further been (anecdotally) described to, “bleat”, “brrr”, “burst”, “cough”, “growl”, “grunt”, “low” “moan”, “moo”, “sneeze”, “snore” or “snort” [23, 25–28]. The snort seems to be the most commonly heard vocalization and has been documented in varying contexts such as being alarmed, annoyed, or when approaching each other [10, 29]. Snorts and bursts are broad-band signals with no harmonic structure (and thus no measurable fundamental frequency); they seem to be produced by a sudden burst of air out of the nostrils [10]. During 700 h of vocal recordings (by day) of giraffes in three zoological institutions, Hurgitsch [10] recorded 72 vocalizations, mostly snorts.

Apart from this, no acoustic descriptions have been provided for the different types of vocalizations listed above. This makes it impossible to assess whether these are different call types or whether the authors used different terms for similar sounds.

It has been suggested (again rather anecdotally) that giraffes do communicate using infrasonic vocalizations (the signals are verbally described to be similar—in structure and function—to the low-frequency, infrasonic “rumbles” of elephants) [27, 30]. It was further speculated that the extensive frontal sinus of giraffes [31] acts as a resonance chamber for infrasound production. Moreover, particular neck movements (e.g. the neck stretch) are suggested to be associated with the production of infrasonic vocalizations.

Despite these reports of giraffe sounds, there is no clear evidence that giraffes indeed use acoustic signals to communicate with each other [32]. Acoustic communication describes the interchange of information between at least two individuals, where an acoustic signal (typically a vocalization) is being directly transmitted from a sender and perceived by a receiver, that alters the behaviour of the communicating animals [33, 34]. Although grunts and snorts are produced in agonistic interactions [28, for personal observation for a grunting adult female giraffe see Additional file 1], it is unclear what role the acoustic signals play compared to the visual, tactile and olfactory cues.

In general, Artiodactyla are highly vocal. Acoustic behaviour and vocalizations of several species have been intensively studied, showing that acoustic cues have a functional relevance in reproductive contexts. Examples include the saiga *Saiga t. tatarica* [35], the red deer *Cervus elaphus* [36] and the North American bison *Bison bison* [37]. Acoustic signals are also important for mother–infant recognition, such as in the goat *Capra hircus* [38], cattle *Bos taurus* [39], sheep *Ovis aries* [40], eland antelope *Taurotragus oryx*, red deer *Cervus elaphus*, reindeer *Rangifer tarandus*, mule deer *Odocoileus hemionus*, white-tailed deer *Odocoileus virginianus* and the pronghorn *Antilocapra americana* [41]. Hurgitsch [10], however, who recording during a birth at Vienna Zoo and later on recorded the mother–calf unit regularly (during daytime), did not document vocal communication between the giraffe mother and her offspring, which is highly uncommon for mammals.

We aim at further investigating vocal communication in giraffes in more detail and collected data of captive individuals during day and night. We particularly focussed on detecting tonal, infrasonic or sustained vocalizations.

**Fig. 1:**
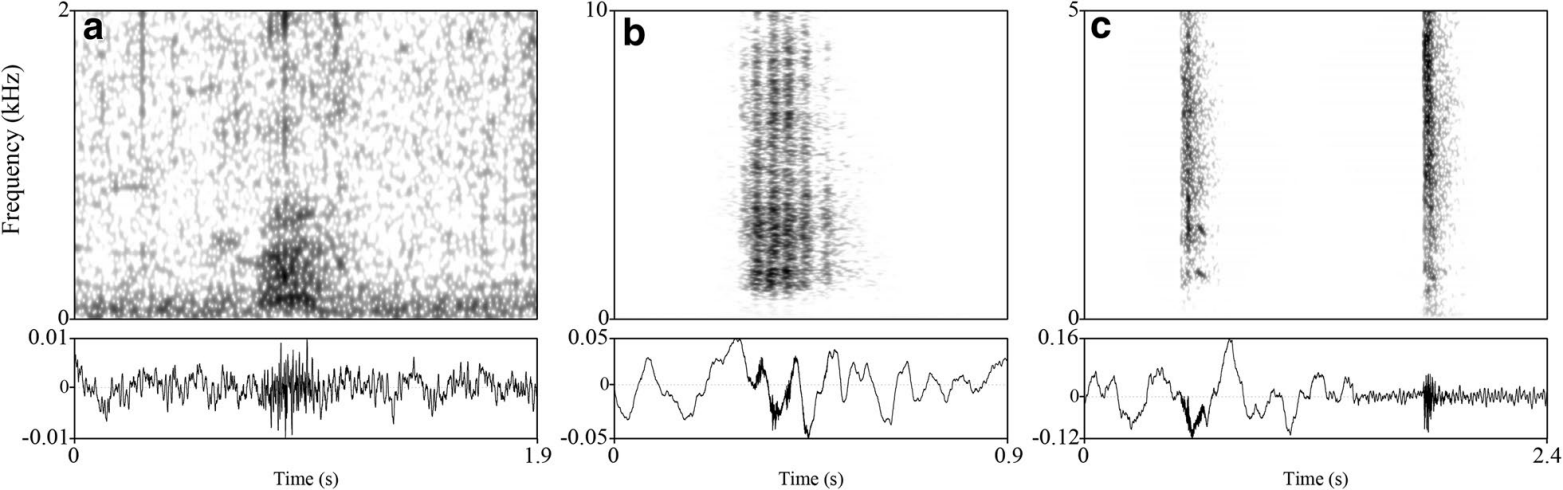
Broad-band spectrograms and waveforms of three giraffe call types. Examples of vocalizations are given for a Grunt (**a**), Snort (**b**) and two Bursts (**c**). The spectrograms were generated in Praat 5.4.01 using the following settings: Gaussian window shape; frequency steps: 1000; time steps: 250; frequency range: 0–10 kHz; window lengths: 0.1 Hz; dynamic range: 40 dB. A sound file for each spectrogram is provided online at the Additional files: **a** (Additional file 2),** b** (Additional file 3),** c** (Additional file 4)

As expected, exploring giraffe vocal communication turned out to be time consuming, tedious and very challenging. Nevertheless, this report presents data indicating that giraffes do produce structurally interesting humming vocalizations apart from the short broadband snorts, bursts and grunting sounds (see Fig. 1a–c; Additional files 2, 3, 4). These hums, however, are apparently mainly produced at night. Based on spectral characteristics, these newly recognized “humming” vocalizations might be of communicative relevance.

By presenting these calls, which have not been acoustically described elsewhere, we want to encourage acoustic research in giraffes. However, we strongly suggest developing an automatic system that helps analysing great amount of acoustic data and related behavioural contexts in order to instigate more research on giraffe vocal behaviour.

## Methods

### Data collection

Data were collected over several months in three European zoos: Berlin Tierpark in Germany, Copenhagen Zoo in Denmark and Vienna Zoo, Austria. We conducted nocturnal indoor and diurnal outdoor recordings. During both recording conditions no other animal species were housed together with the giraffes. The animals had access to the outdoor enclosure throughout the zoo opening hours depending on season and weather condition at all 3 institutions. Giraffe indoor facilities cover the following area size: 600 m^2^ at Berlin Tierpark, 120 m^2^ at Copenhagen Zoo and 130 m^2^ at Vienna Zoo. At Berlin Tierpark each individual was kept in its individual stall overnight. Giraffes at Copenhagen Zoo are generally kept together during the night, but during data collection one pregnant female was separated from the rest of her herd (indoor stall can be divided into 2–4 separate compartments if required). During data collection at the giraffe barn at Vienna Zoo, the giraffe bull was separated from its group during the night.

**Fig. 2:**
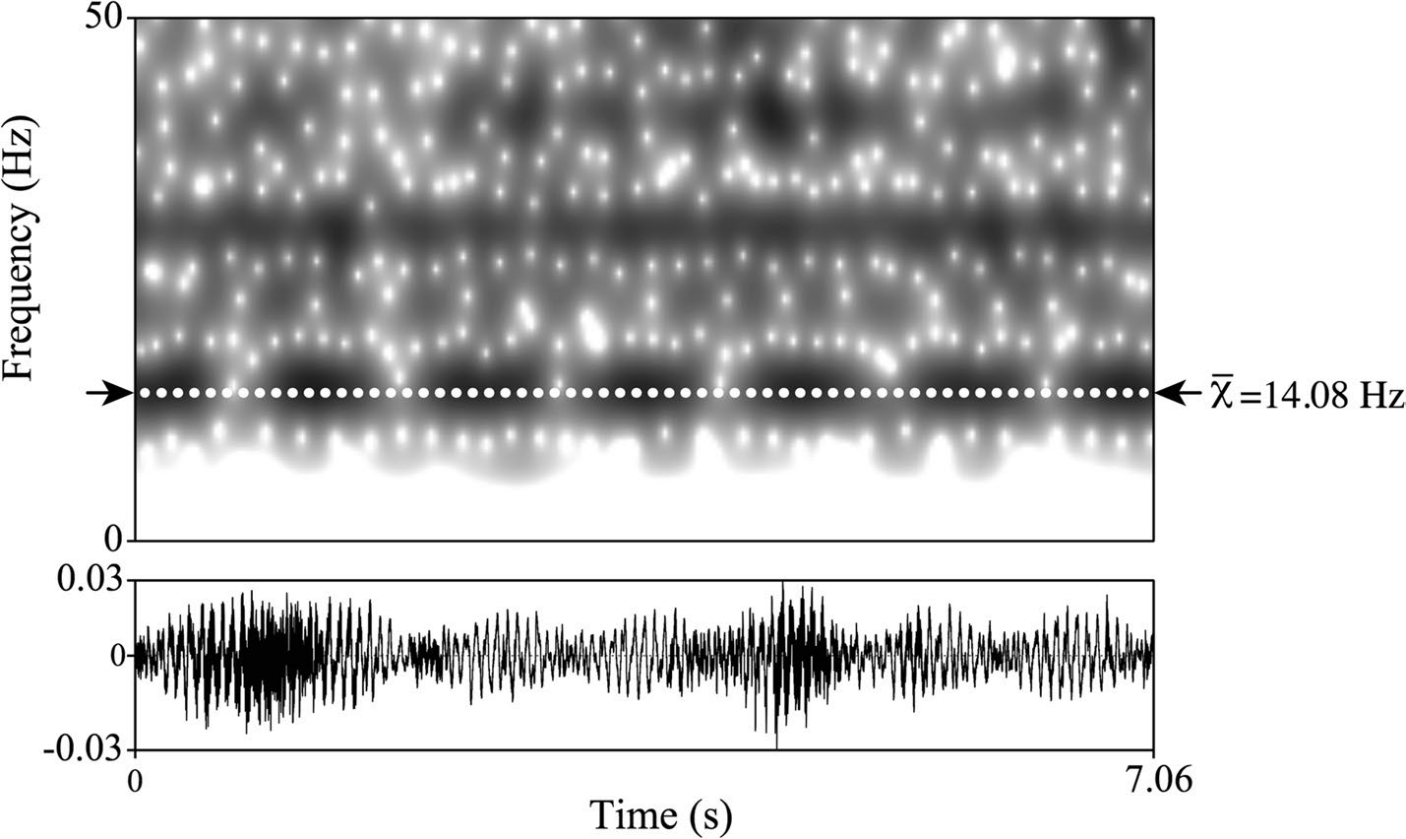
Narrow-band spectrogram and waveform of an acoustic signal within the infrasonic range. Example of an acoustic signal recorded at Berlin Tierpark proving that the used Song Meter SM2+ (Wildlife Acoustics Inc.) for this study is capable of recording frequencies below 20 Hz. *Dashed white line* and *black arrows* indicate a detected signal at an average of 14.08 Hz. The spectrogram was generated in Praat 5.4.01 using the following settings: Gaussian window shape; frequency steps: 1000; time steps: 250; frequency range: 0–10 kHz; window lengths: 0.4 Hz; dynamic range: 40 dB

Outdoor recordings were captured at Berlin Tierpark in October 2011 using a portable Sound Devices 722 audio recorder (frequency response: 10 Hz–40 kHz, +0.1/−0.5 dB) with a sampling rate of 48 kHz and an amplitude resolution of 16 bits. To ensure recordings of possible infrasonic vocalizations, we used an omni-directional custom-built Neumann KM 183 microphone that was modified for recording frequencies below 20 Hz (flat recording down to 5 Hz) and used in previous studies to record infrasonic elephant vocalizations [42, 43]. To capture behaviour during vocal events at the outdoor facility, we used a Samsung CMX-C10R camcorder. Indoor recordings (at night) were conducted using a Song Meter SM2+ digital audio field recorder (Wildlife Acoustics Inc.) equipped with two omni-directional microphones centrally positioned at a height of 3 m. This recording unit has been proven to be effective in recording frequencies in the infrasonic range (a spectrogram from a sound recording from Berlin Tierpark is provided in Fig. 2). The recorder was programmed to record from 6 p.m. to 7 a.m. the next day. Due to missing nocturnal video recordings, however, it was impossible to identify the calling individual or behavioural indicators for sound production [1]. Table 1 lists the year of data collection, total recording time for each institution and number of giraffes including age according to age class categorization by Le Pendu et al. (newborn: <6 months; young: 6–18 months; subadult: 18 months to 4 years; adult: >4 years) [44].

**Table 1:** Number of giraffes including age (year-month), year of data collection and total recording time for each institution

Study site	Giraffe species	Year, month	N	Males				Females				Rec time (hh:mm:ss)
				Adult	Subadult	Young	Newborn (months)	Adult	Subadult	Young	Newborn	
Berlin Tierpark	*Giraffa c. rothschildi*	2011, Oct	11	21 Years 5 months		1 Year 1 month	2	24 Years 5 months	1 Year 8 months			135:21:39
							2	12 Years 2 months				
								9 Years 4 months				
								8 Years 2 months				
								4 Years 7 months				
								4 Years 2 months				
		2012, Nov	9	22 Years 6 months		1 Year 3 months		13 Years 3 months			5 Months	109:10:00
								10 Years 5 months			4 Months	
								9 Years 3 months				
								5 Years 8 months				
								5 Years 3 months				
		2014, Apr	8		2 Years 9 months		5	14 Years 9 months				336:01:24
							0	11 Years 11 months				
								10 Years 8 months				
								7 Years 2 months				
								6 Years 8 months				
Copenhagen Zoo	*Giraffa c. reticulata*	Mar, 2014	7	7 Years 11 months			4	7 Years 7 months	1 Year 9 months	8 Months		107:58:53
								7 Years 8 months				
								10 Years 8 months				
Vienna Zoo	*Giraffa c. rothschildi*	Apr, 2014	4	20 Years 9 months		9 Months		17 Years				144:24:17
								8 Years 6 months				
		Oct − Dec, 2007	4	14 Years 3 months			1	8 Years 6 months				114:16:13
								2 Years				

Age classification based on Le Pendu et al. [44]: newborn: <6 months; young: 6–18 months; subadult: 18 months to 4 years; adult: >4 years

### Acoustic analysis

We visually inspected 908 h and 50 min of nocturnal, and 38 h and 22 min of diurnal recordings for tonal, infrasonic or sustained signals and annotated 65 calls using sound spectrograms (fast Fourier transform method; Gaussian window shape; window lengths: 0.02 s; time steps: 1000; frequency steps: 250; dynamic range: 35–40 dB) generated in PRAAT 5.4.01 DSP package [45]. We did not inspect and listen to short broad noise-bands (and thus putative bursts or snorts) because these are very similar to most other ambient cracking and bumping sounds). Annotated calls were extracted to separate WAV sound files and analysed in a custom-written semi-automatic tool in Matlab [following 42]. This enabled tracing the contour of the fundamental frequency (F0) in the spectrogram. To compute a Fourier spectrogram for the frequency range of 0–800 Hz we used a frame size of 30 ms and a step size of 3 ms.

We extracted the following frequency-related parameters: F0 contour value at the beginning (F0 start) and middle (F0 mid) of each call, and value at the F0 contour offset (F0 end) in hertz (Hz); median (F0 median), lowest (F0 min) and highest (F0 max) frequency value, difference between F0 min and F0 max (F0 range), and calculated average frequency across a call (F0 mean) in Hz. The total duration of each call was used as a temporal parameter. All acoustic features (Table 2) were exported into a comma-separated file, which forms the input for statistical analysis.

**Table 2:** List of 11 acoustic parameters and their definitions

Acoustic parameters	Definition
F0 start (Hz)	Fundamental frequency at the beginning of the vocalization
F0 mid (Hz)	Fundamental frequency at the middle of the vocalization
F0 end (Hz)	Fundamental frequency at the end of the vocalization
F0 min (Hz)	Lowest fundamental frequency
F0 max (Hz)	Highest fundamental frequency
F0 range (Hz)	Difference between minimum and maximum fundamental frequency
F0 mean (Hz)	Arithmetic average frequency across a call
F0 median (Hz)	Central point from data points in ascending order of the F0 contour
Duration (s)	Time between onset to the end of call

### Statistical analysis

Statistical analyses were performed using SPSS software version 22 [46]. We conducted descriptive statistics to obtain general information about the recorded vocalizations by examining the arithmetic averages and standard deviations for all acoustic parameters.

## Results

**Fig. 3:**
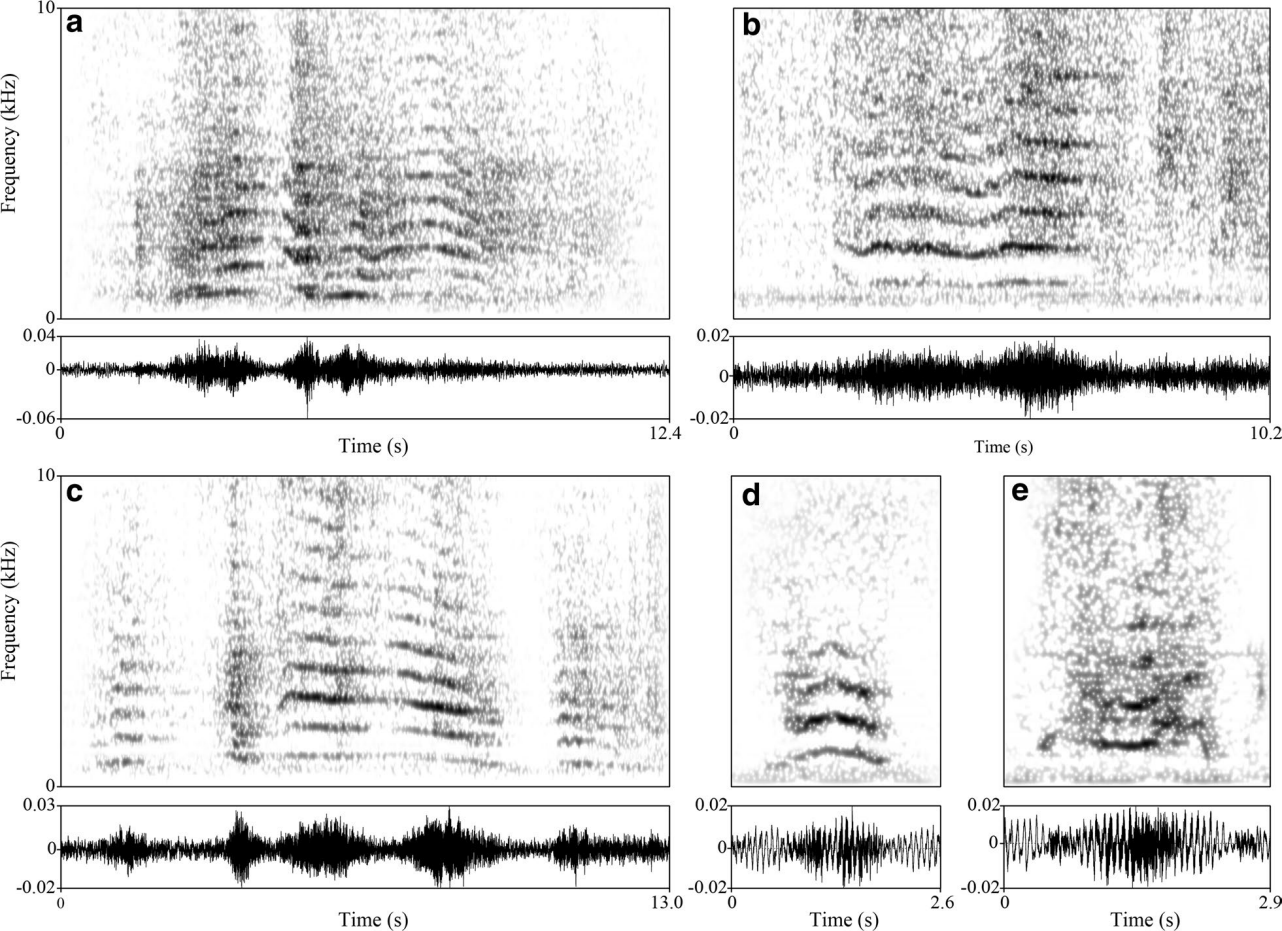
Narrow-band spectrograms and waveforms of five different variations of giraffe humming vocalizations. Examples of five hums differing in acoustic structure and temporal characteristics are given. Spectrograms were generated in Praat 5.4.01 using the following settings: Gaussian window shape; frequency steps: 1000; time steps: 250; frequency range: 10 kHz; window lengths: 0.1 Hz; dynamic range: 35 dB. A sound file for each spectrogram is provided online at the Additional files:** a** (Additional file 5),** b** (Additional file 6),** c** (Additional file 7),** d** (Additional file 8),** e** (Additional file 9)

We recorded 65 humming vocalizations: 34 hums at Berlin Tierpark, 9 hums at Vienna Zoo, and 22 hums at Copenhagen Zoo. Hums were rich in harmonic structure, having a deep and sustained sound with an average fundamental frequency of 92.01 ± 25.78 Hz. Minimum frequencies went down to 35.01 Hz and hums ranged from a minimum length of 0.41 s to a maximum of 4.17 s. This call type was recorded only during the nocturnal recording sessions. Figure 3a–e (Additional files 5, 6, 7, 8, 9) represents spectrograms of different hums. Table 3 provides average values ± standard deviation, minimum and maximum values for the extracted acoustic parameters from each humming. Table S1 (Additional file 10) lists temporal distributions for each analysed “humming” as well as data on sunrise and sunset for each study site.

**Table 3:** Arithmetic mean, standard deviation, minimum and maximum for acoustic parameters extracted from giraffe humming vocalizations

Acoustic parameter	Berlin Tierpark			Copenhagen Zoo			Vienna Zoo		
	N hums = 34			N hums = 22			N hums = 9		
	±	Min	Max	±	Min	Max	±	Min	Max
F0 start (Hz)	82.54 ± 29.32	40.0	145.02	109.8 ± 27.54	40.01	155.03	100.57 ± 40.81	45.01	185.02
F0 mid (Hz)	76.48 ± 18.81	45.01	180.04	110.02 ± 27.04	70.01	180.04	93.35 ± 29.05	50.01	130.01
F0 end (Hz)	73.9 ± 19.41	40.01	185.04	110.7 ± 34.21	65.01	185.04	87.23 ± 35.98	50.01	165.02
F0 min (Hz)	63.98 ± 18.23	35.01	105.02	83.88 ± 20.64	40.01	125.03	77.23 ± 27.4	40.01	125.01
F0 max (Hz)	94.69 ± 23.44	55.01	150.02	143.67 ± 29.61	85.02	195.04	116.68 ± 39.05	60.01	185.02
F0 range (Hz)	30.71 ± 14.59	10.0	76.01	59.79 ± 29.83	15.0	125.03	39.45 ± 21.57	10.0	80.01
F0 mean (Hz)	79.25 ± 18.52	48.53	120.36	110.26 ± 22.07	76.13	160.12	95.6 ± 31.64	52.71	144.69
F0 median (Hz)	78.98 ± 18.67	50.01	116.01	108.77 ± 22.56	75.02	160.03	94.18 ± 30.26	55.01	140.01
Duration (s)	1.42 ± 1.04	0.41	4.17	0.95 ± 0.36	0.45	1.74	1.52 ± 0.87	0.42	3.21

## Discussion

At present, a systematic assessment of giraffe vocal behaviour is missing. Up to now the only scientifically documented giraffe vocalizations are atonal snorts or bursts through the nostrils [10, 32]. One reason for this is that, compared to other social-living mammals, giraffes seem very taciturn.

In this study we analysed hundreds of hours of acoustic recordings from captive giraffes in three institutions and documented a sound that has never been structurally described in the scientific literature before—the “hum”. Although we could not identify the calling individuals, the giraffes definitely produced the recorded sounds because we documented similar vocalizations in three different institutions without any additional co-housing species.

The “hum” is a low-frequency vocalization with a rich harmonic structure and of varying duration. Since it was not possible to determine the calling individual, we are currently unable to prove that this sound is indeed used for communication or to give information about the behavioural context and prospective information content. Although we cannot provide behavioural data, we would like to note that at all 3 zoos all giraffes where kept under similar housing conditions during night times. At Copenhagen Zoo the pregnant cow was separated from her herd, while at Vienna Zoo the giraffe bull was kept separate from the rest. Berlin Tierpark kept each giraffe in an individual stall, however calves where kept together with their mothers. At Copenhagen Zoo hums occurred approximately within 2 h before sunrise, while at the other two zoos, hums occurred mainly in the middle of the night. These patterns might provide suggestive hints that in giraffe communication the “hum” might function as a contact call, for example, to re-establish contact with herd mates.

Nonetheless, the rich harmonic structure and the frequency modulation indicate that this type of vocalization has the potential to convey relevant information to receivers.

Interestingly, these vocalizations have so far been recorded only at night. Even giraffe keepers and zoo managers stated that they have never heard these vocalizations before. Anatomical investigations indicate that giraffes have excellent vision with potentially long-range visual acuity, which would provide a means of communication between widely separated conspecifics [47]. Recent social behaviour research has shown that giraffes spend a significant portion of their vigilance towards social partners [48], suggesting that perception and utilization of visual communication cues are highly developed in the giraffe communication system. Giraffes might use vocalizations more often once vision is limited (e.g. at night time). Future studies should test in a well established experimental setting whether giraffes are more vocal when visual communication cues are absent.

We found no evidence for giraffe infrasonic communication in our data set even though it is widely assumed that giraffes communicate in this manner. The lack of systematic assessment, detailed spectrographic descriptions and presentations or sound examples of giraffe infrasonic signals have not prevented researchers from suggesting adaptive explanations (e.g. keeping vocal contact) or from accepting as fact the idea that giraffes produce infrasound (via Helmholtz resonance, not vocal fold production) to communicate [23, 30–32]. We concede that giraffes in captivity, housed within the same enclosure, might not need to use infrasonic signals to communicate (such signals may be used mainly for long-distance communication when vision is eliminated). Still, neither Bashaw 2003 [32], nor Hurgitsch 2011 [10], nor we could find evidence for giraffe infrasonic communicative signals. Accordingly, such communication in giraffes should remain in a mere hypothesis status.

The relationship between average fundamental frequency and vocal fold length is inversely proportional [49]. Considering that vocal fold tissue behaves like a simple string, the following equation for vibrating strings can be used to determine F0:

$$F0 = \frac{1}{2L}\sqrt {\frac{\sigma }{\rho }} ,$$

where L is the length of the vocal folds in meters, σ is the longitudinal stress (tension) applied to the vocal folds in kPa and *ρ* is the tissue density (1.02 g/cm^3^) [50, 51]. An increase in F0 is correlated with higher stress on the vocal folds [49]. Accordingly, the absence of stress should theoretically yield the lowest possible F0 [following 52]. Based on this assumption and applying the above formula, the minimum producible fundamental frequency for a 41.45 mm giraffe vocal fold [21] would be 12 Hz, well in the infrasonic range (theoretically, therefore, giraffes should be able to produce infrasound with the larynx via passive vocal fold vibration). If we, however, use an empirical model based on the co-variation of vocal fold length and mean F0 across mammals [52], the predicted mean F0 for a 41.45 mm vocal fold should be around 50 Hz (which corresponds reasonably to the mean F0 of the lowest vocalizations recorded in our study).

**Fig. 4:**
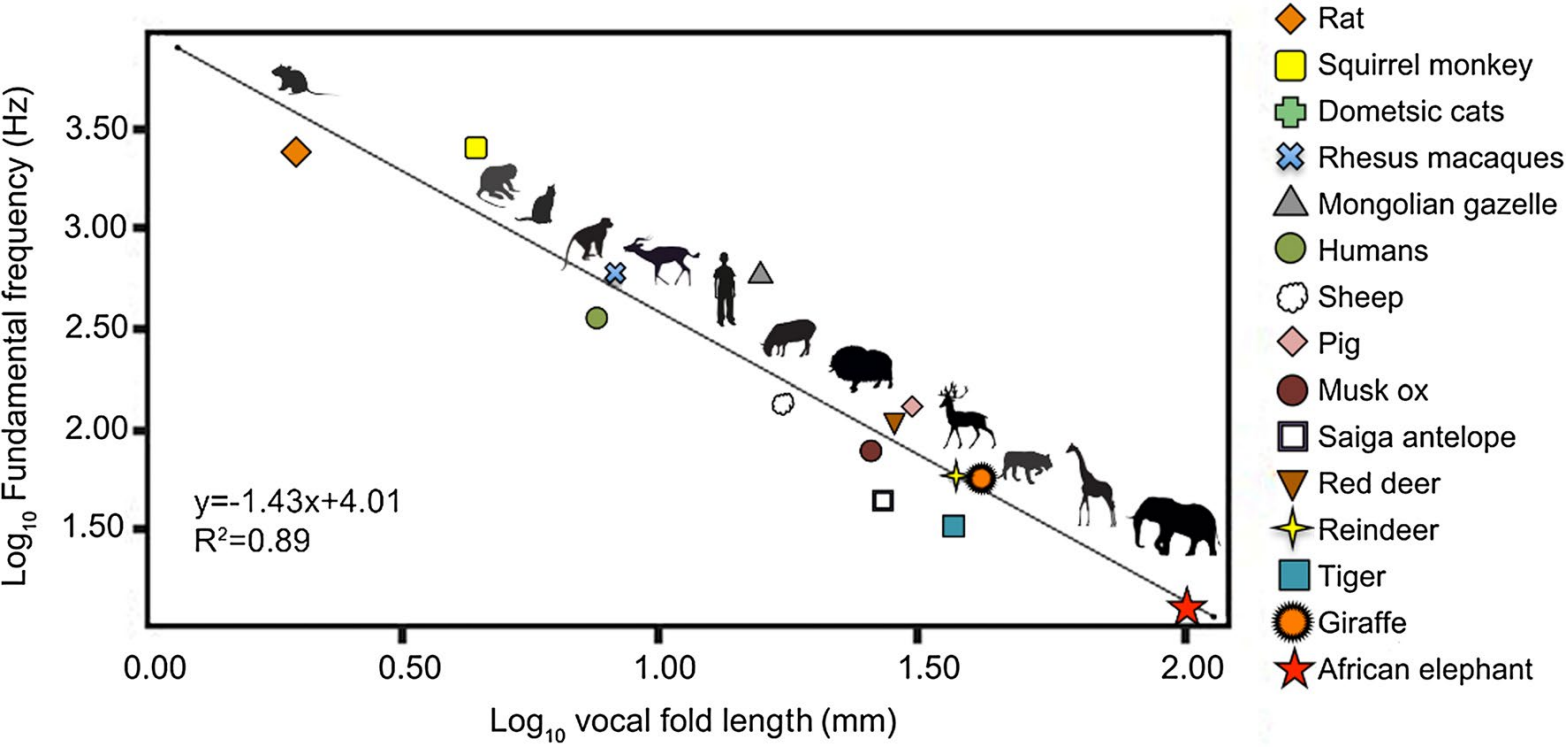
Log-log plot of average F0 for various mammals (n = 15) versus vocal fold length. The linear model predicts that the giraffe’s 41.45 mm [21] vocal fold should result in a mean F0 of ~50 Hz (log_10_ F0 (Hz) = −1.43 × log_10_ vocal fold length (mm) + 4.01). The mean fundamental frequency (indicated by the orange sun) of the recorded giraffe humming vocalizations was 92.01 ± 25.78 Hz, ranging from 35 to 144 Hz. (adapted from Fletcher [53] and Charlton et al. [52]). Data were taken from: [11, 35, 49, 50, 54–66]

Based on the acoustic structure and the theoretical acoustic calculation, the humming vocalizations we recorded could have been produced via passive vocal fold vibration (Fig. 4).

To re-emphasize, our measurements and the calculations mentioned above give no information about identity, vocal tract length and age class of the caller. In general, vocalizations can be used for transferring various information about, for example, individuality, age, gender, arousal, dominance hierarchies or reproductive states [67]. In this study, however, due to absent behavioural data during acoustic recordings, we are unable to make any statement about the context-specific use, or the potential active or passive communicative role of humming.

Another more inherent issue in regards to call nomenclature is worth mentioning. The use of specific terms for giraffe vocalizations in earlier reports [23, 25] were based on the calls’ phonetics and authors’ subjective sound perception, respectively, and not due to comparative and quantitative methods to objectively describe distinct types of vocalizations. Classification of animal sounds requires comparative analyses among individuals; there is, however, no general agreement on how to best categorize calls [68]. The humming vocalizations presented in this study might be the same type of vocalization as one of those reported earlier, but missing acoustic recordings from these reports hinder objectively comparing the data.

The present findings emphasize that vocalizations should be taken into account when studying giraffe social and communicative behaviour. At the same time, we draw attention to the fact that detecting giraffe vocalizations is intricate and time consuming. Clearly, it would be even more difficult to record vocalizations of free-ranging giraffes. This makes zoos or sanctuaries optimal sites for initial exploration. A next step for future studies should be to develop an automatic acoustic monitoring/detecting system linked with concurrent video recordings. This would enable detecting, annotating and recording vocalizations along with the corresponding behaviour and help identify the calling individual.

Furthermore, another possibility to examine vocalizations and potential infrasound production (though behaviourally invasive) in giraffes could be by separating a giraffe spatially from its herd during the night (when visual stimuli are absent and individuals can barely see or locate each other). In addition, playback experiments would help reveal whether giraffes show behavioural reactions in response to conspecific vocalizations.

These approaches could yield novel insights into giraffe vocal behaviour.

### Ethics statement

This non-experimental research meets all applicable international, national and/or institutional guidelines for the care and use of animals. The nature of the study was purely observational: No invasive methodologies were applied at any point of the study. Berlin Tierpark, Copenhagen Zoo and Vienna Zoo approved data collection for the study. All procedures were in accordance with European Union law.

## Additional files

**Additional file 1.** Video of an adult female giraffe chasing an infant male away while grunting at it.
**Additional file 2.** Grunt, audio sample of Fig. 1a.
**Additional file 3.** Snort, audio sample of Fig. 1b.
**Additional file 4.** Bursts, audio sample of Fig. 1c.
**Additional file 5.** Hum, audio sample of Fig. 3a.
**Additional file 6.** Hum, audio sample of Fig. 3b.
**Additional file 7.** Hum, audio sample of Fig. 3c.
**Additional file 8.** Hum, audio sample of Fig. 3d.
**Additional file 9.** Hum, audio sample of Fig. 3e.
**Additional file 10: Table S1.** Temporal distributions of giraffe humming vocalizations from nocturnal acoustic recordings.

## Abbreviation

F0: fundamental frequency
