# Supplementary material for: Nocturnal “humming” vocalizations: adding a piece to the puzzle of giraffe vocal communication
Source: BMC Res Notes. 2015 Sep 9;8:425. doi: 10.1186/s13104-015-1394-3 (PMC4565008; doi:10.1186/s13104-015-1394-3)
Supplement: Supplementary file 10 — Additional file 10: Table S1. Temporal distributions of giraffe humming vocalizations from nocturnal acoustic recordings. [file 13104_2015_1394_MOESM10_ESM.pdf]

Supplementary Table

Nocturnal “humming” vocalizations: adding a piece to the puzzle of giraffe vocal communication

Anton Baotic, Florian Sicks and Angela S. Stoeger

Table S1 Temporal distributions of giraffe humming vocalizations from nocturnal acoustic recordings at three European zoos. Given for each analysed call are date (dd.mm.yy) and time of occurrence during data collection. Times for sunrise and sunset for each date are based on the current GPS location of each institution and were gathered from www.sunearthtools.com. All times (hh:mm) are given in CET (Central European Time)

Study site	Call nr.	Date	Time	Sunrise	Sunset
Copenhagen Zoo	1	11.03.2014	04:00	06:36	18:05
Copenhagen Zoo	2	11.03.2014	04:00	06:36	18:05
Copenhagen Zoo	3	11.03.2014	04:00	06:36	18:05
Copenhagen Zoo	4	11.03.2014	04:00	06:36	18:05
Copenhagen Zoo	5	11.03.2014	04:11	06:36	18:05
Copenhagen Zoo	6	11.03.2014	04:11	06:36	18:05
Copenhagen Zoo	7	11.03.2014	04:11	06:36	18:05
Copenhagen Zoo	8	11.03.2014	04:15	06:36	18:05
Copenhagen Zoo	9	11.03.2014	04:16	06:36	18:05
Copenhagen Zoo	10	11.03.2014	04:55	06:36	18:05
Copenhagen Zoo	11	11.03.2014	04:55	06:36	18:05
Copenhagen Zoo	12	11.03.2014	04:55	06:36	18:05
Copenhagen Zoo	13	11.03.2014	04:55	06:36	18:05
Copenhagen Zoo	14	11.03.2014	04:55	06:36	18:05
Copenhagen Zoo	15	11.03.2014	04:56	06:36	18:05
Copenhagen Zoo	16	11.03.2014	04:56	06:36	18:05
Copenhagen Zoo	17	16.03.2014	04:22	06:23	18:15
Copenhagen Zoo	18	16.03.2014	04:22	06:23	18:15
Copenhagen Zoo	19	16.03.2014	04:23	06:23	18:15
Copenhagen Zoo	20	16.03.2014	04:23	06:23	18:15
Copenhagen Zoo	21	16.03.2014	04:23	06:23	18:15
Copenhagen Zoo	22	16.03.2014	04:24	06:23	18:15

Berlin Tierpark	23	23.06.2012	02:30	04:43	21:33
Berlin Tierpark	24	24.06.2012	21:23	04:44	21:33
Berlin Tierpark	25	24.06.2012	21:23	04:44	21:33
Berlin Tierpark	26	24.06.2012	04:06	04:44	21:33
Berlin Tierpark	27	24.06.2012	04:06	04:44	21:33
Berlin Tierpark	28	25.06.2012	23:32	04:44	21:33
Berlin Tierpark	29	25.06.2012	01:07	04:44	21:33
Berlin Tierpark	30	25.06.2012	01:08	04:44	21:33
Berlin Tierpark	31	27.06.2012	00:22	04:45	21:32
Berlin Tierpark	32	27.06.2012	01:16	04:45	21:32
Berlin Tierpark	33	28.06.2012	01:29	04:45	21:32
Berlin Tierpark	34	28.06.2012	01:29	04:45	21:32
Berlin Tierpark	35	28.06.2012	01:29	04:45	21:32
Berlin Tierpark	36	28.06.2012	01:29	04:45	21:32
Berlin Tierpark	37	29.06.2012	00:50	04:46	21:32
Berlin Tierpark	38	29.06.2012	00:55	04:46	21:32
Berlin Tierpark	39	29.06.2012	01:48	04:46	21:32
Berlin Tierpark	40	07.10.2011	04:09	07:16	18:30
Berlin Tierpark	41	11.10.2011	06:03	07:23	18:21
Berlin Tierpark	42	06.05.2014	02:55	05:25	20:40
Berlin Tierpark	43	06.05.2014	02:55	05:25	20:40
Berlin Tierpark	44	13.04.2014	03:55	06:13	20:00
Berlin Tierpark	45	13.04.2014	03:18	06:13	20:00
Berlin Tierpark	46	13.04.2014	07:03	06:13	20:00
Berlin Tierpark	47	16.04.2014	01:20	06:06	20:06
Berlin Tierpark	48	18.04.2014	23:19	06:02	20:09
Berlin Tierpark	49	20.04.2014	01:40	05:57	20:13
Berlin Tierpark	50	23.04.2014	00:13	05:51	20:18
Berlin Tierpark	51	26.04.2014	01:37	05:45	20:34
Berlin Tierpark	52	29.04.2014	02:05	05:38	20:28
Berlin Tierpark	53	04.05.2014	01:51	05:29	20:37
Berlin Tierpark	54	05.05.2014	00:00	05:27	20:38
Berlin Tierpark	55	06.05.2014	22:28	05:25	20:40
Berlin Tierpark	56	07.05.2014	22:21	05:23	20:42
Vienna Zoo	57	02.10.2007	20:28	06:54	18:33
Vienna Zoo	58	02.10.2007	20:29	06:54	18:33

Vienna Zoo	59	03.10.2007	23:33	06:56	18:31
Vienna Zoo	60	03.10.2007	00:32	06:56	18:31
Vienna Zoo	61	03.10.2007	03:16	06:56	18:31
Vienna Zoo	62	27.03.2014	23:53	05:43	18:17
Vienna Zoo	63	27.03.2014	23:53	05:43	18:17
Vienna Zoo	64	26.03.2014	22:30	05:45	18:16
Vienna Zoo	65	05.04.2014	05:28	06:25	19:30
